# Effective Hydrogen Production from Alkaline and Natural Seawater using WO_3__–*x*_@CdS_1–*x*_ Nanocomposite-Based Electrocatalysts

**DOI:** 10.1021/acsomega.3c02516

**Published:** 2023-09-11

**Authors:** Mohamed Jaffer Sadiq Mohamed, Mohammed Ashraf Gondal, Muhammad Hassan, Munirah Abdullah Almessiere, Asif Ali Tahir, Anurag Roy

**Affiliations:** † Laser Research Group, Department of Physics & Interdisciplinary Research Center for Hydrogen and Energy Storage (IRC-HES), King Fahd University of Petroleum and Minerals (KFUPM), Dhahran 31261, Saudi Arabia; ‡ K. A. CARE Energy Research and Innovation Center, King Fahd University of Petroleum and Minerals, Dhahran 31261, Saudi Arabia; § Department of Biophysics, Institute for Research and Medical Consultations (IRMC), Imam Abdulrahman Bin Faisal University, Dammam 31441, Saudi Arabia; ∥ Department of Physics, College of Science, Imam Abdulrahman Bin Faisal University, Dammam 31441, Saudi Arabia; ⊥ Solar Energy Research Group, Environment and Sustainability Institute, Faculty of Environment, Science and Economy, University of Exeter, Penryn Campus, Cornwall TR10 9FE, U.K.

## Abstract

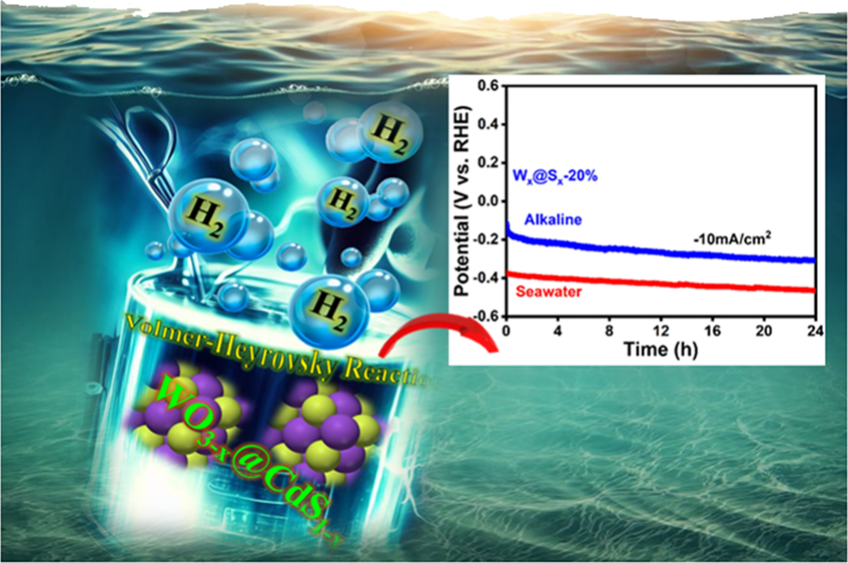

Offshore hydrogen production through water electrolysis presents significant technical and economic challenges. Achieving an efficient hydrogen evolution reaction (HER) in alkaline and natural seawater environments remains daunting due to the sluggish kinetics of water dissociation. To address this issue, we synthesized electrocatalytic WO_3–*x*_@CdS_1–*x*_ nanocomposites (WCSNCs) using ultrasonic-assisted laser irradiation. The synthesized WCSNCs with varying CdS contents were thoroughly characterized to investigate their structural, morphological, and electrochemical properties. Among the samples tested, the WCSNCs with 20 wt % CdS_1–*x*_ in WO_3–*x*_ (W_*x*_@S_*x*_-20%) exhibited superior electrocatalytic performance for hydrogen evolution in a 1 M KOH solution. Specifically, the W_*x*_@S_*x*_-20% catalyst demonstrated an overpotential of 0.191 V at a current density of −10 mA/cm^2^ and a Tafel slope of 61.9 mV/dec. The W_x_@S_x_-20% catalysts demonstrated outstanding stability and durability, maintaining their performance after 24 h and up to 1000 CV cycles. Notably, when subjected to natural seawater electrolysis, the W_*x*_@S_*x*_-20% catalysts outperformed in terms of electrocatalytic HER activity and stability. The remarkable performance enhancement of the prepared electrocatalyst can be attributed to the combined effect of sulfur vacancies in CdS_1–*x*_ and oxygen vacancies in WO_3–*x*_. These vacancies promote the electrochemically active surface area, enhance the rate of charge separation and transfer, increase the number of electrocatalytic active sites, and accelerate the HER process in alkaline and natural seawater environments.

## Introduction

The incessant rise of environmental pollution, climate change, and energy crises has compelled humanity to seek energy resources that possess abundance, affordability, cleanliness, ecological friendliness, and sustainability. In light of this, hydrogen has emerged as a prospective fuel for the future, offering the potential to address various challenges associated with environmental and energy crises.^1,2^ It is established that hydrogen can efficiently be produced at low cost via the electrolysis of water, offering a versatile resource of renewable energy like solar and wind.^3^ Research revealed that the alkaline electrocatalytic hydrogen evolution reaction (HER) is preferable with acidic and neutral electrolytes because of less corrosion for equipment and transition metal-based electrodes. Compared to acidic electrolytes, when H^+^ is produced in the alkaline medium, an extra dissociation process called the Volmer mechanism (H_2_O + e^–^ → H^+^ + OH^–^) is involved.^4^ Recent research indicates that seawater electrolysis is the most environmentally friendly method of producing hydrogen.^5−7^ It is very challenging to develop high-performance electrocatalysts that can withstand poor conductivity, severe corrosivity, poisoning influence, and poor long-term stability.^8^ So far, despite an outstanding hydrogen evolution activity of platinum (Pt)-based electrocatalysts owing to almost zero Gibbs free energy, the scarcity and high cost of Pt limits their widespread applications.^9^ To surmount this shortcoming, intensive research efforts have been made to develop highly stable, durable, nontoxic, efficient, and low-cost alkali metal-based HER electrocatalysts, including various oxides, sulfides, phosphides, carbides, and hydroxides, in the past decades.^10−21^

Lately, tungsten trioxide (WO_3_) has generated renewed interest toward energy conversion and storage due to its flexible band gap, strong electron acceptability, abundance, environmental amiability, mild corrosion resistance, and stability against water.^22^ However, properties such as low intrinsic conductivity and inadequate exposure to active sites of WO_3_ limit its electrocatalytic application. To resolve these issues, various strategies such as the dopant inclusion, electrochemically active surface area (ECSA) widening, morphology modification, phase engineering, vacancy generation, and heterostructure formation have been adopted. The main idea of all these studies was to improve the electrical structures and active sites at the surfaces, interfaces, and edges.^23,24^ Based on this fact, the current work intends to improve the electrical traits of WO_3_ by activating vacancies and defects in its inert basal planes.

Solid surface chemistry imparts distinctive physicochemical qualities (such as optical properties and electrical conduction) to WO_3,_ beneficial for many practical applications.^25^ WO_3_ is frequently found in substoichiometric compositions, denoted as WO_3-*x*_, where 0 < *x* < 1. The rapid reduction of oxygen in these compositions leads to oxygen vacancies (OVs) forming when oxygen atoms are released onto the metal oxide surfaces.^26^ These OVs, which become rich in localized electrons, account for the unique physicochemical properties of WO_3-*x*_. Such properties include optical characteristics, electron transport, charge carrier separation, and surface texture. Consequently, this results in a high number of active sites suitable for catalysis.^27,28^

Due to its high electrical conductivity, the semiconducting inorganic transition-metal chalcogenide, like cadmium sulfide (CdS), has been used as a strong electrocatalyst.^29^ Introducing sulfur vacancies into CdS can create more active sites and enhance the charge-transfer rates, leading to excellent electrocatalytic performance.^30,31^ As a result, CdS has emerged as a captivating candidate for coupling with WO_3_ in the HER context. It can be inferred that the exceptional stability of CdS in solid-aqueous environments can enhance the corrosion resistance and electrocatalytic activity of WO_3_-encapsulated CdS nanocomposites during the HER process. Enhancing the catalytic activity per unit area is imperative to achieve a highly efficient electrocatalyst for the HER. Several factors contribute significantly to the superior performance of effective electrocatalysts, including improved solid electrical conduction to facilitate electron transfer, reduced dimensions (particularly at the nanoscale level) to increase exposure of active sites, porous morphology to promote reactant and product diffusion, and a large flat surface area.^1,32,33^

To the best of our knowledge, a comprehensive review of recent literature indicates a limited number of studies investigating the effectiveness of WO_3_-incorporated CdS as a photocatalyst for hydrogen production.^34−41^ Furthermore, no studies have been conducted to explore their potential as electrocatalysts for HER catalytic activity. Additionally, the influence of S–O dual active vacancies on the crystal facets of WO_3__–*x*_@CdS_1–*x*_ nanocomposites (WCSNCs) as electrocatalysts for HER has not been investigated in the literature. This study presents the first report on synthesizing a series of WCSNCs using an ultrasonic-assisted laser irradiation method with varying WO_3–*x*_ to CdS_1–*x*_ concentration ratios. The results demonstrate that the HER performance of the proposed WCSNC-based electrocatalyst in alkaline and natural seawater media can be tailored by controlling the number of S and O vacancies in the nanocrystalline facets.

## Experimental Section

### Materials

Pure chemical reagents of sodium tungstate (Na_2_WO_4_·2H_2_O) and cadmium chloride (CdCl_2_·2.5H_2_O), sodium sulfide (Na_2_S·9H_2_O), sodium borohydride (NaBH_4_), potassium hydroxide (KOH), Nafion solution (5 wt %), ethanol (C_2_H_5_OH), and hydrochloric acid (HCl of 36–38%) were purchased from Sigma-Aldrich and used without further treatment. Millipore’s Autopure technology was used to get deionized water (DIW).

### Synthesis of WO_3–*x*_ Nanosheets

**Scheme 1:**
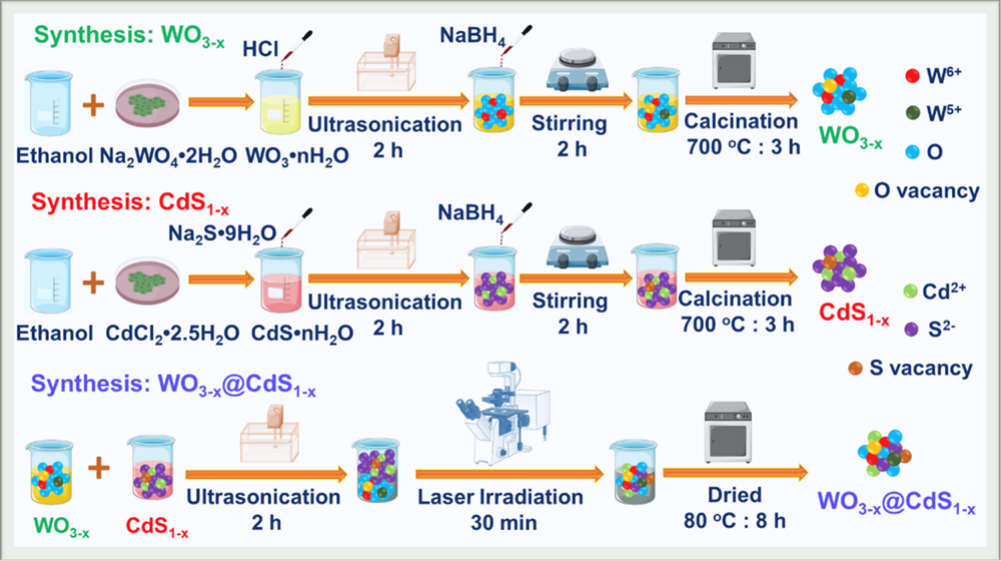
Schematic Representation of the Synthesis of WO_3__–*x*_, CdS_1–*x*_, and W_*x*_@S_*x*_-20%

The WO_3__–*x*_ nanosheets were synthesized by using an ultrasonic process according to Scheme 1. Initially, 20 mL of HCl was combined with 100 mL of 1.0 M Na_2_WO_4_·2H_2_O in ethanol, and the mixture was subjected to sonication for 2 h. Subsequently, the resulting suspension was stirred at room temperature for 6 h. A dropwise addition of 1 mL of 1.0% NaBH_4_ solution was performed, followed by an additional 2 h of stirring. Following this procedure, the suspension was washed with DIW and dried for 8 h at 80 °C, forming WO_3__–*x*_ nanosheets. Finally, the dried powder was crushed and subjected to calcination at 700 °C for 3 h, after which it was allowed to cool to ambient temperature naturally, producing WO_3__–*x*_ nanosheets.

### Synthesis of CdS_1–*x*_ Nanospheres

The synthesis of CdS_1–*x*_ nanospheres was performed by using an ultrasonic process (Scheme 1). Initially, 228 mg of CdCl_2_·2.5H_2_O was combined with 100 mL of ethanol in a beaker. The resulting mixture was vigorously stirred for 6 h, and then 1 mL of 0.1% NaBH_4_ was added dropwise. Subsequently, the mixture was sonicated for 30 min to ensure thorough solution mixing. Following 2 h of ultrasonic processing, the addition of 78 mg of Na_2_S·9H_2_O caused an instant change in the color of the solution to yellow. Finally, the suspension was washed with distilled water and dried at 80 °C for 8 h to obtain the desired CdS_1–*x*_ nanospheres.

### Synthesis of WO_3__–*x*_@CdS_1–*x*_ Nanocomposites

The WO_3__–*x*_@CdS_1–*x*_ nanocomposites were synthesized by using a simple ultrasonic-assisted laser irradiation technique (Scheme 1). In this procedure, different mixtures of WO_3__–*x*_ and CdS_1–*x*_ were prepared with the following percentages: WO_3__–*x*_/*Y* wt % CdS_1–*x*_ (*Y* = 5, 10, 20, and 30%). A homogenized mixture of 5 mg of CdS_1–*x*_ and 100 mg of WO_3-x_ was taken to prepare WO_3__–*x*_/5 wt % CdS_1–*x*_. The CdS_1–*x*_ powder was dispersed separately in DIW for 1 h and mixed with WO_3__–*x*_ powder, followed by sonication for another 1 h. Each mixture was placed and irradiated by a laser for 30 min. The postsynthesis purification was unnecessary because no catalysts or intermediary chemicals were used to complete the laser irradiation procedure. Consequently, the synthesized products were pure without any contaminants. The prepared WCSNCs were named as W_*x*_@S_*x*_-5% (WO_3__–*x*_/5 wt % CdS_1–*x*_), W_*x*_@S_*x*_-10% (WO_3__–*x*_/10 wt % CdS_1–*x*_), W_*x*_@S_*x*_-20% (WO_3__–*x*_/20 wt % CdS_1–*x*_), and W_*x*_@S_*x*_-30% (WO_3__–*x*_/30 wt % CdS_1–*x*_) depending on the concentration of CdS_1–*x*_.

### Characterizations

All sample characterizations were conducted under ambient conditions. Crystal structures of the samples were determined using X-ray diffraction (XRD) analysis performed on a Rigaku D XRD instrument equipped with a Cu Kα line of wavelength (λ = 0.154 nm). The instrument was operated at 40 kV and 40 mA. The microstructures and morphology of the prepared WCSNCs electrocatalysts were examined by using scanning electron microscopy (SEM) with a JEOL instrument and transmission electron microscopy (TEM) with a JEM-2100F instrument. Elemental analyses were carried out using energy-dispersive X-ray spectroscopy with the SEM machine, which provided spectra and maps of the samples. X-ray photoelectron spectroscopy (XPS) spectra were recorded by using a Thermo Scientific K-Alpha instrument with an Al-Kα line operated at 15 kV and 15 mA. The C 1s peak at 284.8 eV was used as a reference, and the pass binding energy was set at 30 eV for analysis.

### Electrochemical Measurements

The electrochemical characteristics of the obtained catalysts were assessed by using a typical three-electrode setup on an AutoLab electrochemical workstation. The active working electrodes modified on a glassy carbon (GC) surface are prepared as follows: WCSNCs (4 mg) and 5 wt % Nafion solution (50 μL) was added to C_2_H_5_OH (950 μL), and then the resultant suspension was ultrasonicated for 20 min to generate a homogeneous catalyst ink. Later, the obtained catalyst ink (5 μL) was drop-cast onto the GC layer (mass loading ∼ 0.285 mg/cm^2^). After 12 h of drying at room temperature, the catalyst-modified GC surface served as a working electrode. The KCl-saturated Ag/AgCl and graphite rods were the reference and counter electrodes. Using the Nernst relation (*E*_RHE_ = *E*_Ag/AgCl_ + 0.0592 pH + 0.1989 V), all potentials measured against a Ag/AgCl electrode were used to convert reversible hydrogen electrodes (RHE). Before the test, pure N_2_ gas was purged into the solution constantly for about 30 min to eliminate oxygen. The polarization curves of the catalysts (immersed in a KOH solution of 1.0 M at pH 13.5) were recorded by linear sweep voltammetry (LSV of scan rate 10 mV/s). The polarization curves were used to generate the Tafel graphs. The resistance of the samples against charge transport in the range of 10^5^ and 10^–1^ Hz was examined using electrochemical impedance spectroscopy (EIS). The samples’ electrochemical double-layer capacitance (*C*_dl_) in KOH solution (1.0 M) was determined by scanning CV in the scan speed range of 20–120 mV/s in the non-Faradaic region. ECSA = *C*_dl_/*C*_s_ values were evaluated in specific capacitance (*C*_s_ of mean value 35 μF/cm^2^).^42^ Chronopotentiometry measurements were used to examine the stability and durability of the proposed catalysts at 10 mA/cm^2^. In addition, the cycling performance of the proposed electrode was evaluated by repeating LSV for 1000 cycles to measure the catalyst performance.

## Results and Discussion

### XRD Analysis

**Figure 1:**
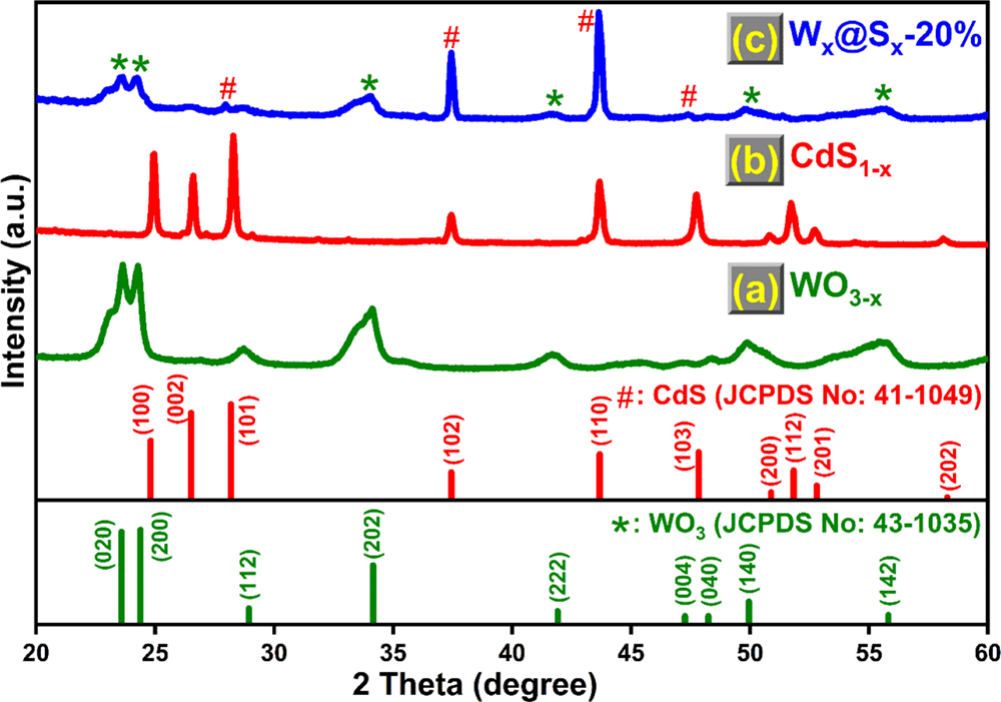
(a–c) Powder XRD patterns of WO_3__–*x*_, CdS_1–*x*_, and W_*x*_@S_*x*_-20% samples.

Figure 1 displays the XRD patterns of various samples. XRD profile of WO_3__–*x*_ (Figure 1a) revealed weak broad peaks indicating a monoclinic phase structure (JCPDS No. 43-1035). XRD profile of CdS_1–*x*_ (Figure 1b) showed a hexagonal phase structure for diffraction peaks (JCPDS No. 41-1049). After loading CdS_1–*x*_ with WO_3__–*x*_, the XRD patterns of W_*x*_@S_*x*_-20% (Figure 1c) are similar to the WO_3__–*x*_ diffraction peak, signifying no disruption in the lattice structures of WO_3__–*x*_ due to the addition of CdS_1–*x*_. The composites were successfully prepared, as shown by the presence of six diffraction peaks at 23.64°, 24.28°, 34.12°, 41.68°, 49.88°, and 55.71°, which correspond to the (020), (200), (202), (222), (140), and (142) planes of WO_3__–*x*_, respectively. In the meanwhile, the presence of four diffraction peaks at 28.28°, 37.44°, 43.68°, and 47.76°, which correspond to the (101), (102), (110), and (103) planes of CdS_1–*x*_ in W_*x*_@S_*x*_-20%, indicates effective coupling of CdS_1–*x*_ with WO_3__–*x*_.

### Morphology Analysis

**Figure 2:**
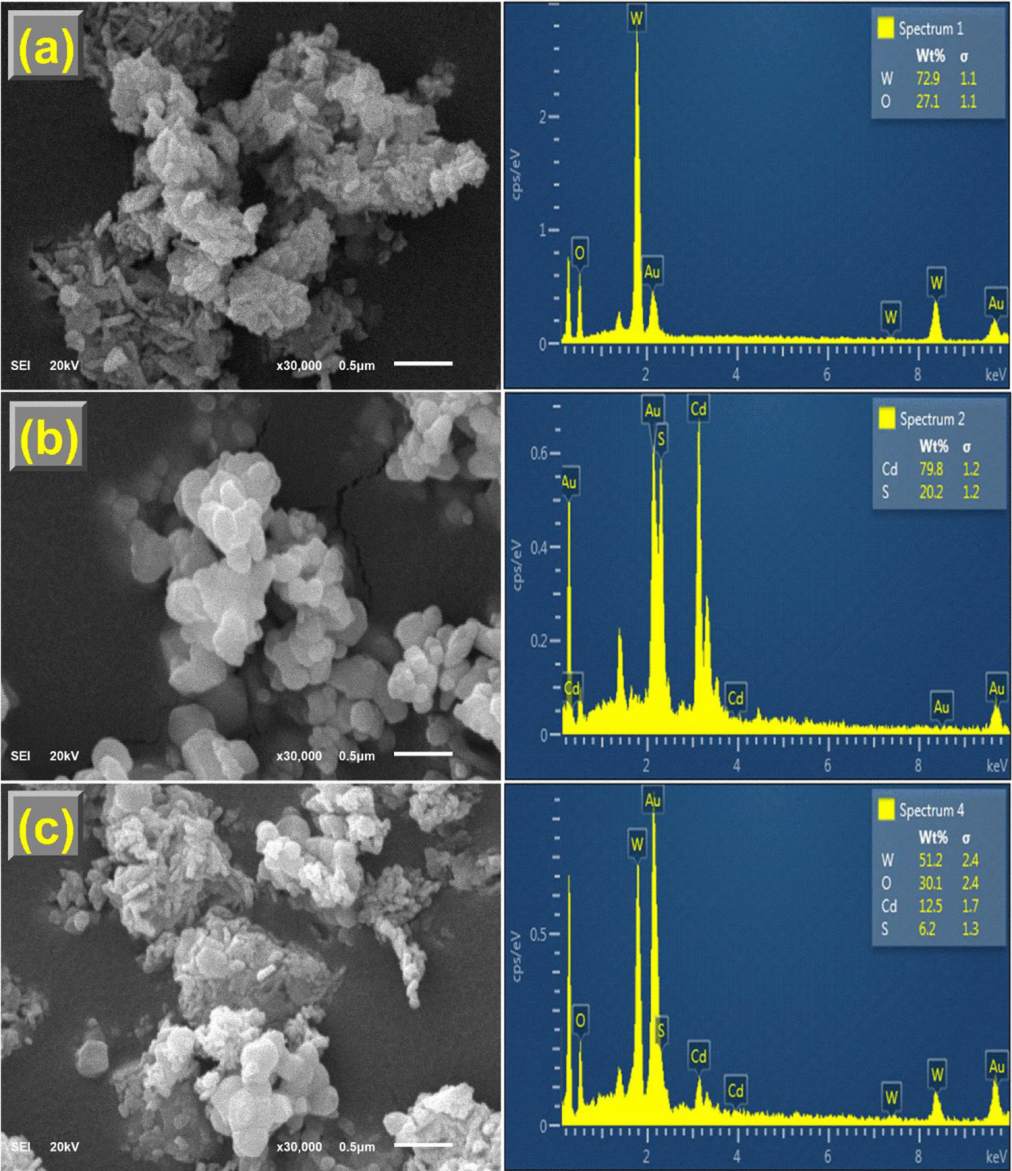
(a–c) SEM images and corresponding EDX spectrum of WO_3__–*x*_, CdS_1–*x*_, and W_*x*_@S_*x*_-20%.

**Figure 3:**
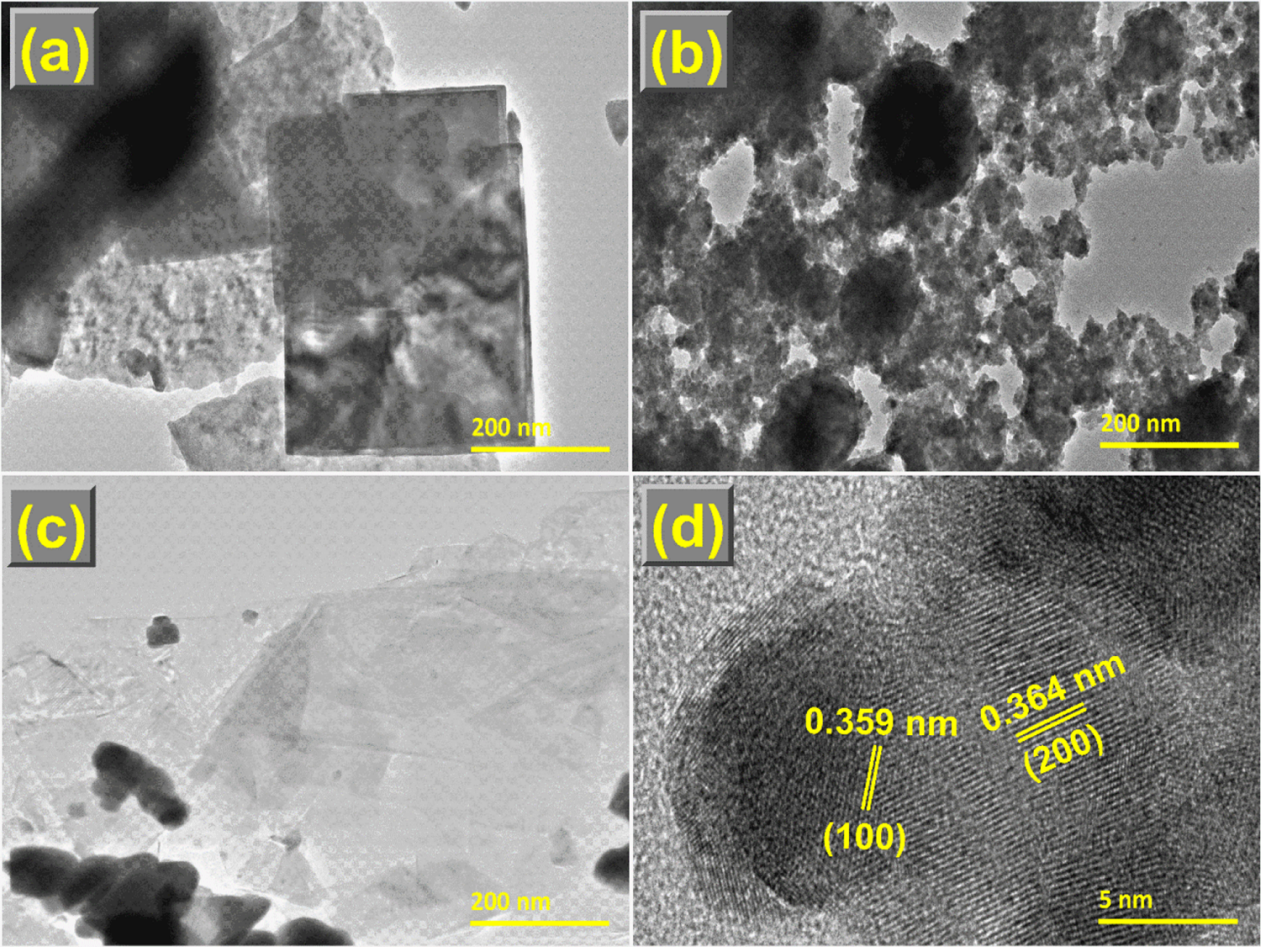
(a–c) TEM bright field images of WO_3__–*x*_, CdS_1–*x*_, and W_*x*_@S_*x*_-20%, respectively. (d) HRTEM image of the W_*x*_@S_*x*_-20% sample.

**Figure 4:**
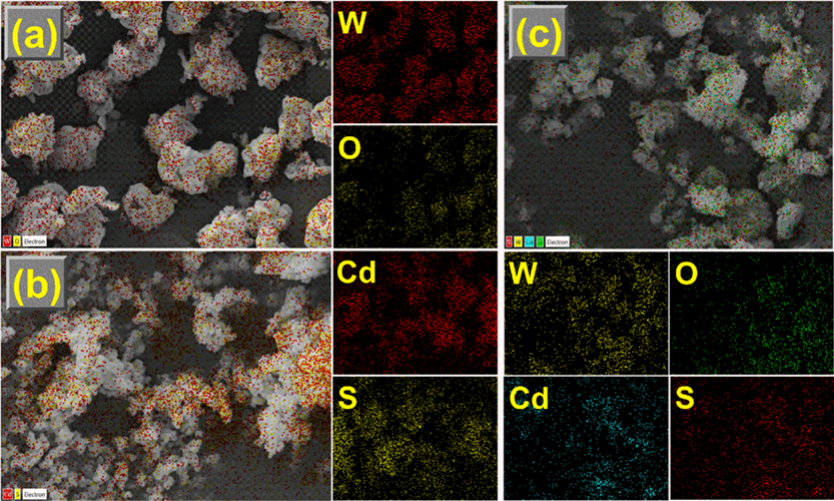
(a–c) Elemental surface color maps of WO_3__–*x*_, CdS_1–*x*_, and W_*x*_@S_*x*_-20% samples.

Figure 2 illustrates the SEM images and the corresponding EDX spectra of WO_3__–*x*_, CdS_1–*x*_, and W_*x*_@S_*x*_-20%. Pure CdS_1–*x*_ (Figure 2b) comprises nanospheres evenly distributed in a monodispersed cluster. The presence of ultrathin nanosheets of WO_3__–*x*_ (Figure 2a) was beneficial for loading CdS_1–*x*_ nanospheres. The CdS_1–*x*_ nanospheres were grown directly on top of WO_3__–*x*_ ultrathin nanosheets while maintaining their original shapes when WO_3__–*x*_ was included in the reaction mixture (Figure 2c). Figures 3a–c and 4a–c display the TEM images and EDX maps of the WO_3__–*x*_, CdS_1–*x*_, and W_*x*_@S_*x*_-20% specimen. The HRTEM image of W_*x*_@S_*x*_-20% (Figure 3d) showed robust connectivity of WO_3__–*x*_ and CdS_1–*x*_ nanoparticles in the matrix. The observed *d*-spacings of WO_3__–*x*_ and CdS_1–*x*_ obtained from the lattice fringes were 0.364 and 0.359 nm, respectively, corresponding to the lattice planes of (200) and (100), respectively. The presence of W, O, Cd, and S in the EDX spectra, further Au peak relating to the use of Au coated grids for the analysis (Figure 2a–c), and elemental mappings (Figure 4a–c) **i**ndicated that CdS_1–*x*_ was strongly coupled to WO_3__–*x*_, which was adequate for significant improvement in the charge transfer between CdS_1–*x*_ and WO_3__–*x*_.

### XPS Analysis

**Figure 5:**
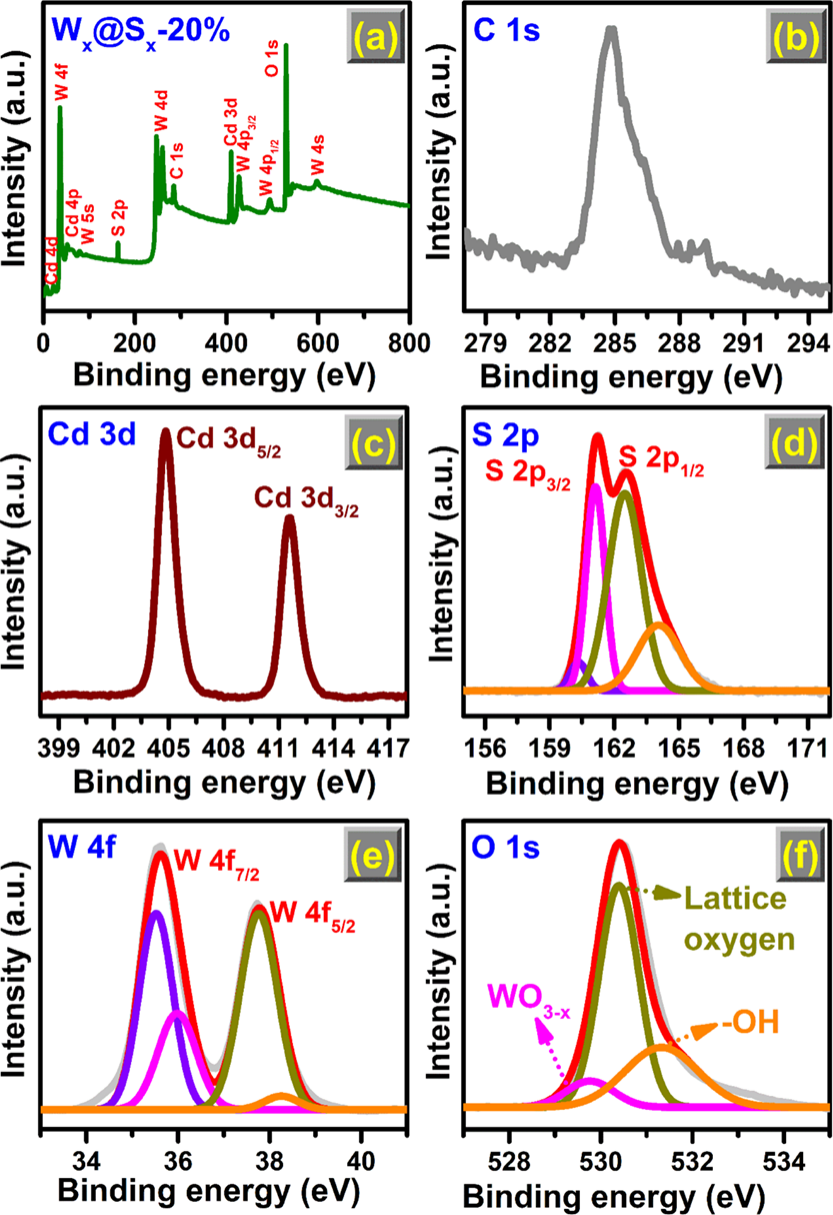
XPS (a) survey spectrum and corresponding core-level spectrum for (b) C 1s, (c) W 4f, (d) Cd 3d, (e) S 2p, and (f) O 1s of the W_*x*_@S_*x*_-20% sample.

The XPS spectrum (Figure 5) was analyzed to identify the surface chemical state of W_*x*_@S_*x*_-20%. The survey spectrum (Figure 5a) detected the elements S, O, Cd, W, and C, wherein C originated from the substrate. The complete absence of other elements in the prepared WCSNCs confirmed its high purity. The binding energy (BE) of the C 1s peak (284.8 eV) was taken as the reference standard (Figure 5b). The two peaks can be seen in the Cd 3d high-resolution spectrum (Figure 5c) at 404.83 and 411.63 eV, which may be attributed to the typical spin–orbit splitting between 3d_5/2_ and 3d_3/2_ of Cd^2+^.^43^Figure 5d shows that the S 2p spectrum exhibited sharp peaks corresponding to 2p_3/2_ and 2p_1/2_ of S at BEs of 161.27 and 162.47 eV, assigned to the metal–sulfur bonds. The existence of the other two peaks was due to the sulfur vacancies.^44^ Two sets of peaks were observed in the 4f spectrum of W (Figure 5e), wherein the peaks for 4f_7/2_ and 4f_5/2_ of W were located at 35.51 and 37.73 eV (corresponding to W^6+^) and the peaks corresponding to 4f chemical states of W ranged from +4 to +6. Forming a substoichiometric version of WO_3__–*x*_ in the form of W^5+^ facilitates shear defects and charge transfer.^45^

Meanwhile, oxygen vacancies are detected by analyzing the O 1s spectra (Figure 5f). Three distinctive peaks in the 1s spectra of O centered at 529.75, 530.40, and 531.33 eV were correspondingly due to oxygen in WO_3__–*x*_, hydroxyl, and lattice oxygen. Three peaks convolve with the O 1s of WO_3__–*x*_, confirming the generation of oxygen vacancies in the proposed WCSNCs.^46^ XPS results also verified the successful blending of CdS_1–*x*_ with WO_3__–*x*_ and the formation of S and O vacancies in the NCs required for HER performance improvement.

### HER Performance

**Figure 6:**
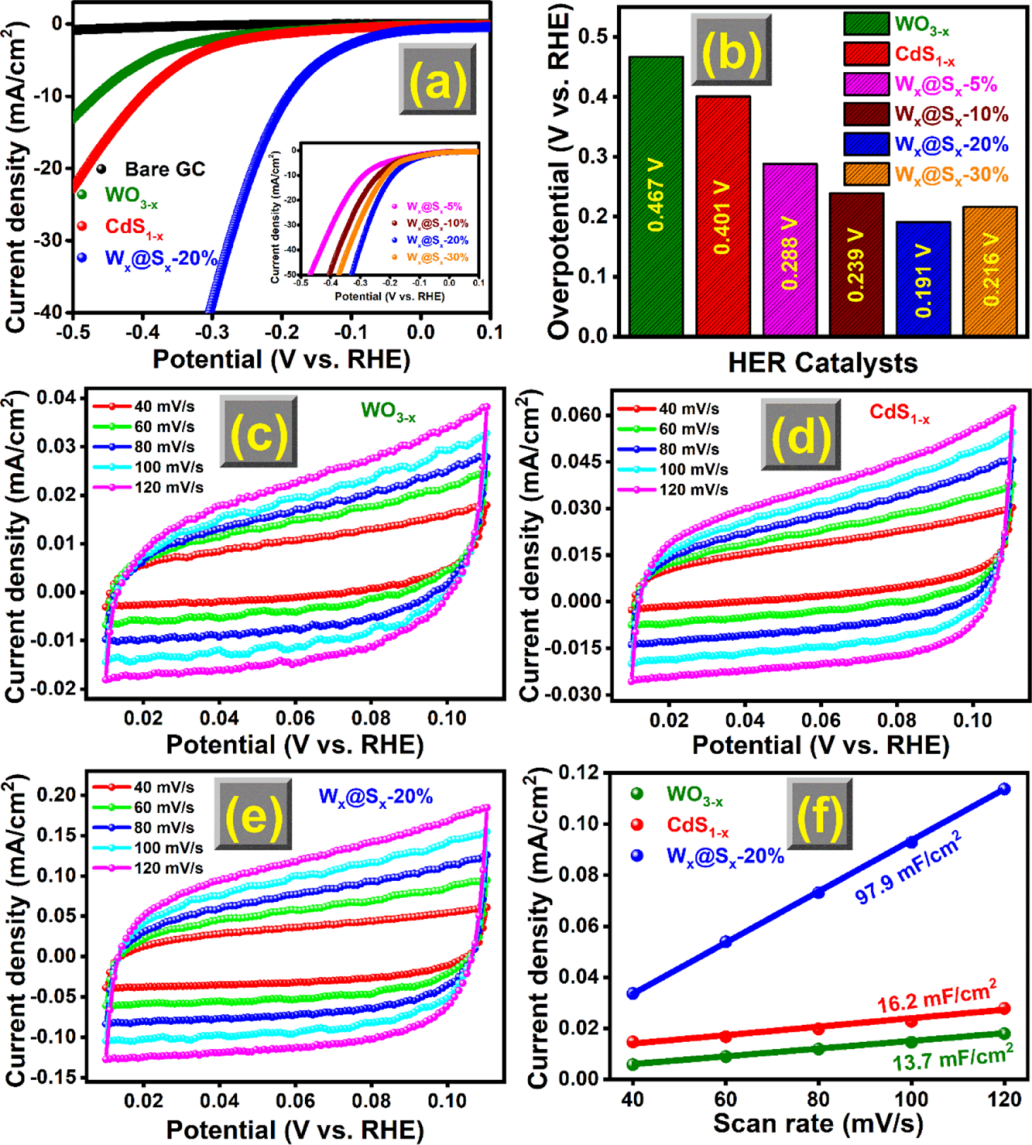
(a) Polarization plots, (b) HER performance, (c, d) CV curves, and (e, f) *C*_dl_ plot of WO_3__–*x*_, CdS_1–*x*_, and W_*x*_@S_*x*_-20%.

Figure 6a–f shows the tested samples’ polarization curves, HER activity (overpotentials at 10 mA/cm^2^), CV curve, and *C*_dl_ plot. W_*x*_@C-20% specimen showed a low overpotential (η_10_ = 0.191 V) and best performance compared to WO_3__–*x*_ (0.467 V), CdS_1–*x*_ (0.401 V), W_*x*_@S_*x*_-5% (0.288 V), W_*x*_@S_*x*_-10% (0.239 V), W_*x*_@S_*x*_-20% (0.191 V), and W_*x*_@S_*x*_-30% (0.216 V) (Figure 6a). However, the HER impact progressively diminishes when the CdS_1–*x*_ concentration exceeds 20 wt %. In addition, the observed increase in the HER overpotential of W_*x*_@S_*x*_-30% to 0.216 V was mainly due to the increase of the CdS_1–*x*_ to WO_3__–*x*_ concentration (the sole active phase) and excess CdS_1–*x*_ that acted as the charge-carrier combination center. In addition to the data presented here, it is worth mentioning that the synergistic impact of WO_3__–*x*_ coupled with CdS_1–*x*_ is responsible for the improved HER catalytic activity of synthesized W_*x*_@S_*x*_-20%. In this study, WO_3__–*x*_ exhibits negligible hydrogen generation activity, most likely because WO_3__–*x*_ must overcome high resistance and weak catalytic activity to achieve the HER criteria. Figure 6b depicts the overpotential histogram bar plot of WO_3__–*x*_, CdS_1–*x*_, and W_*x*_@S_*x*_-20%. Table S1 presents a comprehensive comparison between the synthesized electrocatalyst and other state-of-the-art electrocatalysts in alkaline media. The comparison encompasses a detailed analysis of various key parameters and characteristics, highlighting the strengths and weaknesses of each electrocatalyst.

**Figure 7:**
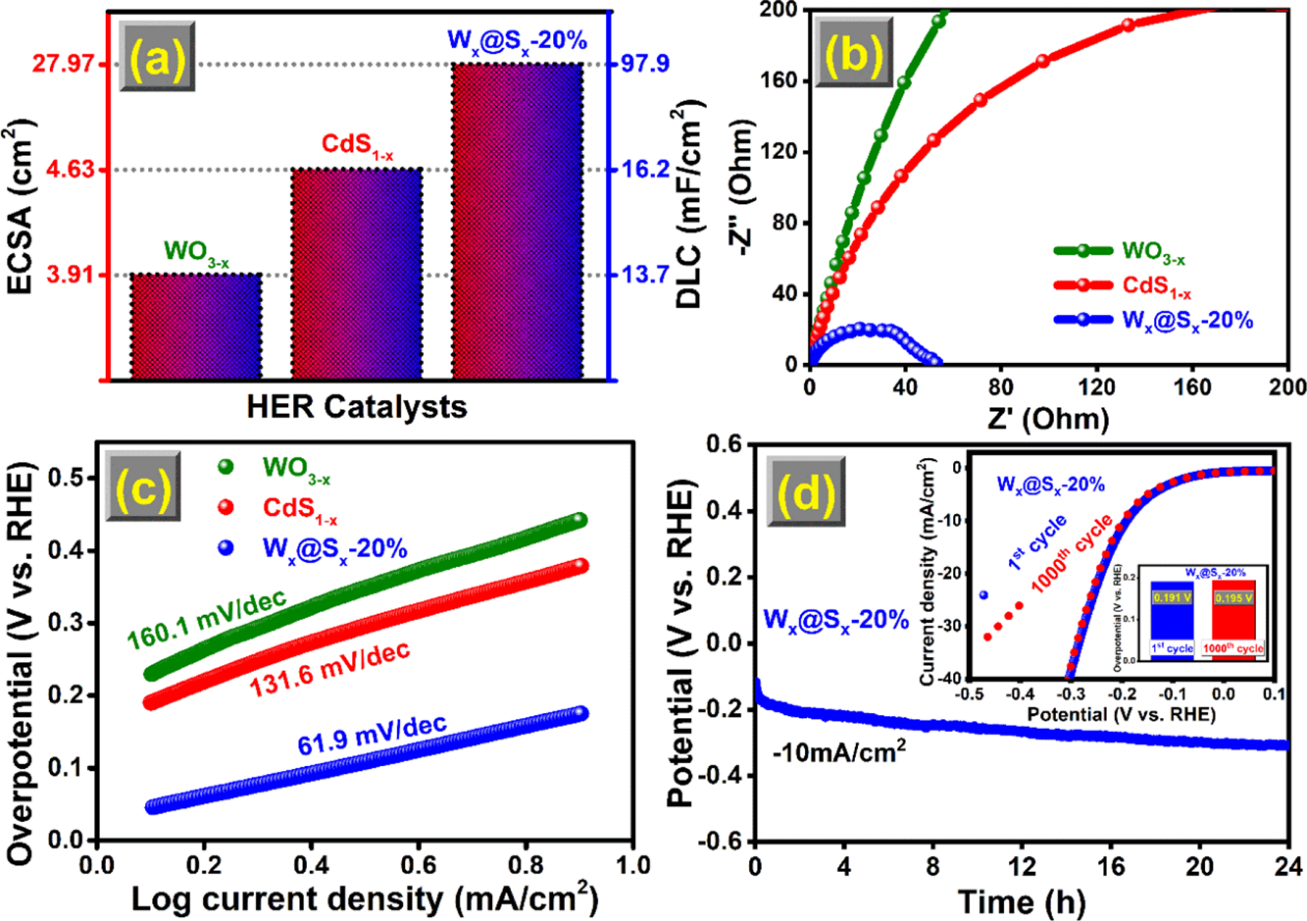
(a) Calculated values of *C*_dl_ and ECSA, (b) Nyquist plots, (c) Tafel plots of WO_3__–*x*_, CdS_1–*x*_, and W_*x*_@S_*x*_-20%, and (d) stability plot after 1000 cycles (inset) and chronopotentiometry plot of W_*x*_@S_*x*_-20%.

Figure 6c–e depicts the CV curves of WO_3__–*x*_ and CdS_1–*x*_ (in basic media), which was used to determine the electrochemical *C*_dl_ values in the non-Faradaic region, estimating the ECSA values of the obtained WCSNC-based catalysts. The *C*_dl_ values (Figure 6f) were computed and presented based on the initial findings. Among the pure samples, the W_*x*_@S_*x*_-20% sample showed the highest *C*_dl_ value of 97.9 mF/cm^2^, about 7.1 times higher than that of bare WO_3__–*x*_ (13.7 mF/cm^2^) and approximately 6.0 times more than that of pure CdS_1–*x*_ (16.2 mF/cm^2^). Consequently, among the prepared WO_3__–*x*_ and CdS_1–*x*_ catalysts, the ECSA of W_*x*_@S_*x*_-20% is the greatest, with a calculated value of 27.97 cm^2^ (Figure 7a). The W_*x*_@S_*x*_-20% sample showed excellent HER activity, affirming the synergistic effects of WO_3__–*x*_ and CdS_1–*x*_ that could boost the intrinsic activity.

EIS spectra of the WCSNC-based catalysts were analyzed to determine the electrochemical kinetic processes responsible for the improved HER activity. The Nyquist plots derived from EIS data are shown in Figure 7b. As seen in Nyquist plots, W_*x*_@S_*x*_-20% has a substantially lower charge-transfer resistance than other samples. It is well understood that a high charge-transport rate corresponds to a low charge-transfer resistance and vice versa. As a result, W_*x*_@S_*x*_-20% may improve HER performance by decreasing the charge-transfer resistance and increasing the charge-transport rate during the electrocatalytic process. The Tafel slopes were used to describe the reaction kinetics of the catalysts, as illustrated in Figure 7c. When compared with WO_3__–*x*_ (160.1 mV/dec) and CdS_1–*x*_ (131.6 mV/dec), the Tafel slope of W_*x*_@S_*x*_-20% (61.9 mV/dec) was lower. The results indicated that the alkaline HER kinetics were the fastest in W_*x*_@S_*x*_-20% catalysts. The HER process can be explained in three stages:^4,47^ discharge (Volmer reaction), electrochemical desorption (Heyrovsky reaction), and Tafel recombination with the corresponding Tafel slopes of 120, 40, and 30 mV/dec. The achieved Tafel slope of W_*x*_@S_*x*_-20% (61.9 mV/dec) was intermediate between the Volmer and Heyrovsky reactions; the Volmer–Heyrovsky mechanism was expected to play a significant role in W_*x*_@S_*x*_-20% for the HER in basic media. The suggested mechanism for HER is based on the above analysis results. The WO_3__–*x*_ surface is superior for H_2_O adsorption and dissociation, while the CdS_1–*x*_ surface performs better for H_ads_ adsorption. These allowed the H released during H_2_O molecule breakdown to be transferred to the CdS_1–*x*_ interface contact region and converted into H_2_.

A suitable catalyst should have both effective HER activity and high stability. Long-term chronopotentiometry (CP) and CV were used to assess the stability of the catalysts. The CP results show the decaying overpotential caused by the continuous HER process over 24 h of W_*x*_@S_*x*_-20% (Figure 7d) and WO_3__–*x*_ (Figure S1). Before and after 1000 CV scans, the polarization curves of W_*x*_@S_*x*_-20% virtually overlapped with each other (inset of Figure 7d). The observed overpotential values at a current density of 10 mA/cm^2^ are 191 mV for the first cycle and 195 mV after 1000 cycles, suggesting only a slight decay in the electrochemical performance after 1000 cycles. It clearly shows that the electrochemical features of W_*x*_@S_*x*_-20% are almost retained even after 1000 cycles. W_*x*_@S_*x*_-20% showed a remarkable HER stability performance in the alkaline medium.

Additionally, an SEM study was conducted on the electrocatalyst after evaluating its stability performance, as shown in Figure S2. The analysis demonstrates the presence of agglomerated catalyst particles that exhibit minimal alteration, indicating a high degree of stability in the electrocatalyst. These findings align consistently with the stability results depicted in Figure 7d.

The achieved improvement in the catalytic activity and stability of W_*x*_@S_*x*_-20% was ascribed to the following reasons: (i) The W_*x*_@S_*x*_-20%, with its ultrathin nanosheets and nanosphere shapes, has a greater electrochemical active surface area, allowing for increased contact between an electrocatalyst and an electrolyte, and by exploiting these active sites, resulting in improved catalytic performance, (ii) the Nyquist plots have a smaller diameter in alkaline media, suggesting reduced resistance during charge transfer and faster electrode kinetics, and (iii) the synergistic impact of dual active oxygen and sulfur vacancies defects in WO_3__–*x*_ and CdS_1–*x*_ crystal facets lead to substantially greater electrocatalytic HER performance of W_*x*_@S_*x*_-20%.

**Figure 8:**
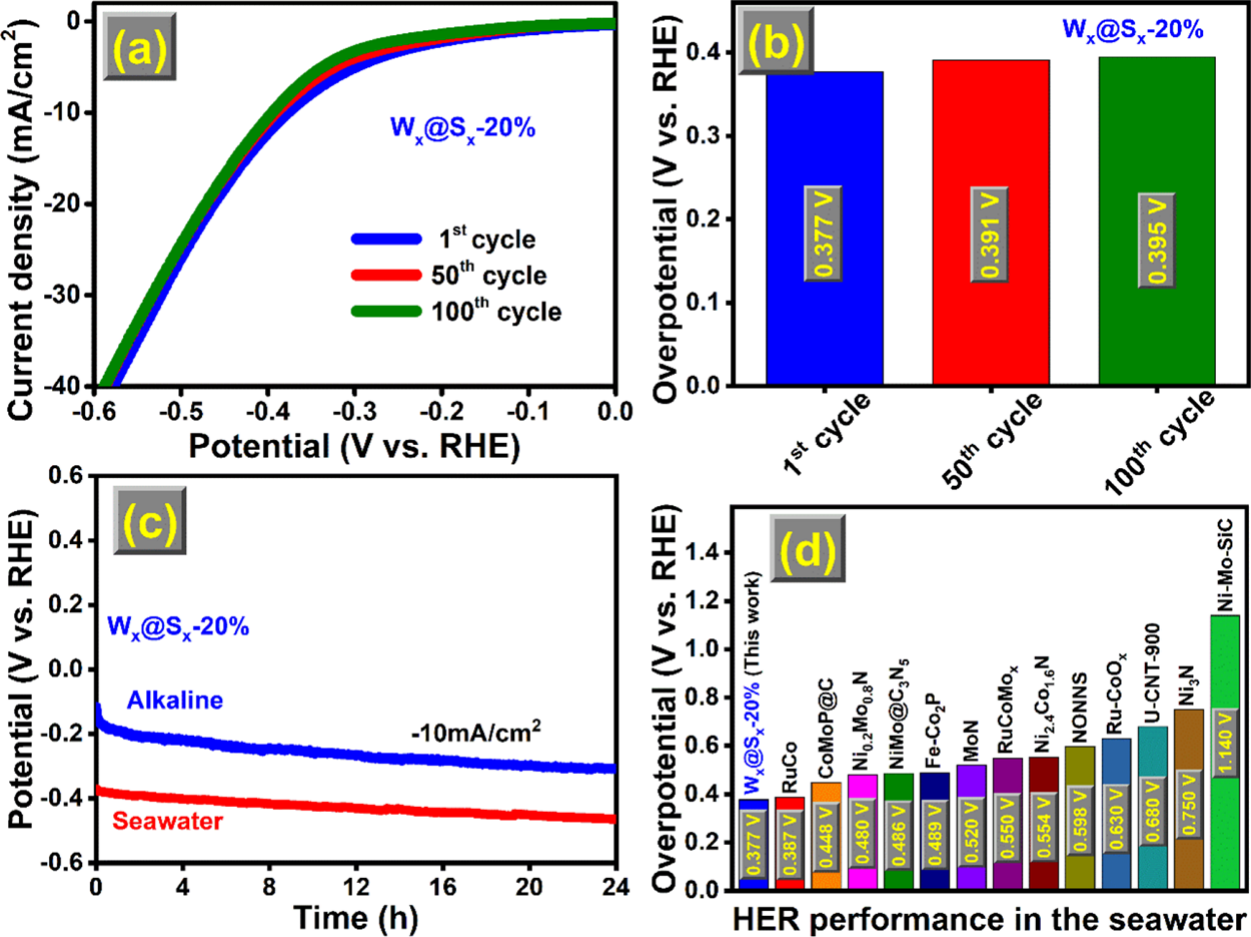
W_*x*_@S_*x*_-20%. (a) LSV curve, (b) overpotential histogram, (c) CP curve, and (d) overpotential comparison with others previously reported in the literature using the same current density of 10 mA/cm^2^ under seawater circumstances.

The direct electrolysis of seawater is an efficient and environmentally friendly method of producing hydrogen energy.^48^ However, considering the ultimate goal of seawater electrolysis, the HER remains a barrier. We assessed the W_*x*_@S_*x*_-20% electrocatalytic HER performance in natural seawater (pH ∼ 8.0, collected on Dammam Beach, Saudi Arabia). Figure 8a illustrates that the LSV polarization curve of W_*x*_@S_*x*_-20% decreases slightly with increasing testing cycle over the first, 50th, and 100th cycles, enabling the overpotential of 0.377, 0.391, and 0.395 V to deliver a current density of 10 mA/cm^2^ and showed higher activity than the commercial 20 wt % Pt/C catalyst under high potentials (>0.749 V vs RHE).^48^Figure 8b depicts the overpotentials of the different W_*x*_@S_*x*_-20% cycles necessary to achieve a current density of 10 mA/cm^2^. Seawater has several chemicals, including ions that may degrade and poison the electrocatalyst. As a result, the stability of the catalyst is critical for long-term electrolysis.^57^ The CP curves revealed that W_*x*_@S_*x*_-20% could maintain activity for 24 h at a current density of 10 mA/cm^2^ (Figure 8c). These findings indicate that W_*x*_@S_*x*_-20% has much promise to produce hydrogen from seawater electrolysis. Significantly, the synthesized W_*x*_@S_*x*_-20% showed higher electrocatalytic activity than the previously reported catalysts^5,6,8,48−56^ as shown in Figure 8d for the natural seawater conditions.

Consequently, the synthesis yielded a heterogeneous interface characterized by the prepared WO_3__–*x*_@CdS_1–*x*_ composite, demonstrating remarkable synergistic catalytic activity. First, the structural properties of WO_3__–*x*_ facilitated the disintegration of water molecules, leading to the liberation of their constituent atoms and subsequent hydrogen formation from water protons. Second, incorporating CdS_1–*x*_ in the composite enhanced the catalyst’s conductivity, facilitating efficient charge transfer. Finally, the WO_3-x_@CdS_1–*x*_ composite exhibited a commendable electrocatalytic performance for the HER in alkaline water and natural seawater environments.

## Conclusions

The present study uses ultrasonic-assisted laser irradiation to synthesize a series of WO_3__–*x*_@CdS_1–*x*_ nanocomposites. The electrocatalytic performance of the optimized nanocomposite (W_*x*_@S_*x*_-20%) as a catalyst for the HER was evaluated under alkaline and natural seawater conditions. XPS analysis confirmed the presence of sulfur (S) and oxygen (O) vacancies in the synthesized electrocatalyst, indicating a strong interaction between WO_3__–*x*_ and CdS_1–*x*_. The best-performing electrocatalyst demonstrated significantly improved HER activity, as evidenced by a lower overpotential (0.191 V) and a smaller Tafel slope (61.9 mV/dec). The Volmer–Heyrovsky mechanism was proposed to account for the enhanced HER performance of W_*x*_@S_*x*_-20%.

Furthermore, the optimized nanocomposite exhibited excellent repeatability and durability in an alkaline environment, demonstrated by its stable performance even after 1000 cycles and 24 h of continuous electrolysis. The introduction of S vacancies in CdS_1–*x*_ and O vacancies in WO_3__–*x*_ resulted in a considerable increase in the ECSA, charge separation, charge-transfer rate, and active sites for electrocatalysis, thus enhancing the HER activity of the catalyst. During natural seawater electrolysis, the W*_x_*@S*_x_*-20% catalyst showed superior HER activity and stability. This study demonstrates that tailoring the active O and S vacancies in the nanocrystalline structures of WO_3__–*x*_ and CdS_1–*x*_ can enhance electrocatalytic HER performance. The prepared electrocatalysts based on WO_3__–*x*_ and CdS_1–*x*_ nanocomposites have promising potential for future energy storage and conversion applications, with the opportunity for further optimization through crystal facet regulation and defect engineering. With systematic and in-depth material research and continuous optimization, hydrogen production through seawater electrolysis is expected to make significant advancements.

## Supporting Information Available

CP curves of WO_3__–*x*_ and W_*x*_@S_*x*_-20% samples; SEM micrographs of the W_*x*_@S_*x*_-20% sample recorded after the stability test; and comparison of the HER activities of various electrocatalysts in the alkaline electrolyte (PDF)
