# Supplementary material for: Effective Hydrogen Production from Alkaline and Natural Seawater using WO3–x@CdS1–x Nanocomposite-Based Electrocatalysts
Source: ACS Omega. 2023 Sep 11;8(37):33332–41. doi: 10.1021/acsomega.3c02516 (PMC10515405; doi:10.1021/acsomega.3c02516)
Supplement: Supplementary file 1 — ao3c02516_si_001.pdf [file ao3c02516_si_001.pdf]

# Supporting Information

## Effective Hydrogen Production from Alkaline and Natural Seawater using $WO_{3-x}$@$CdS_{1-x}$ Nanocomposites-based Electrocatalyst

Mohamed Jaffer Sadiq Mohamed,[1] Mohammed Ashraf Gondal,[1,2]* Muhammad Hassan,[1] Munirah Abdullah Almessiere,[3,4] Asif Ali Tahir,[5] Anurag Roy[5]*

[1]*Laser Research Group, Department of Physics & Interdisciplinary Research Center for Hydrogen and Energy Storage (IRC-HES), King Fahd University of Petroleum and Minerals (KFUPM), Dhahran 31261, Saudi Arabia.*

[2]*K. A. CARE Energy Research and Innovation Center, King Fahd University of Petroleum and Minerals, Dhahran 31261, Saudi Arabia.*

[3]*Department of Biophysics, Institute for Research and Medical Consultations (IRMC), Imam Abdulrahman Bin Faisal University, Dammam 31441, Saudi Arabia.*

[4]*Department of Physics, College of Science, Imam Abdulrahman Bin Faisal University, Dammam 31441, Saudi Arabia.*

[5]*Solar Energy Research Group, Environment and Sustainability Institute, Faculty of Environment, Science and Economy, University of Exeter, Penryn Campus, Cornwall, TR10 9FE, U.K.*

***Corresponding Authors**: magondal@kfupm.edu.sa (M.A.G.); A.Roy30@exeter.ac.uk (A.R.)

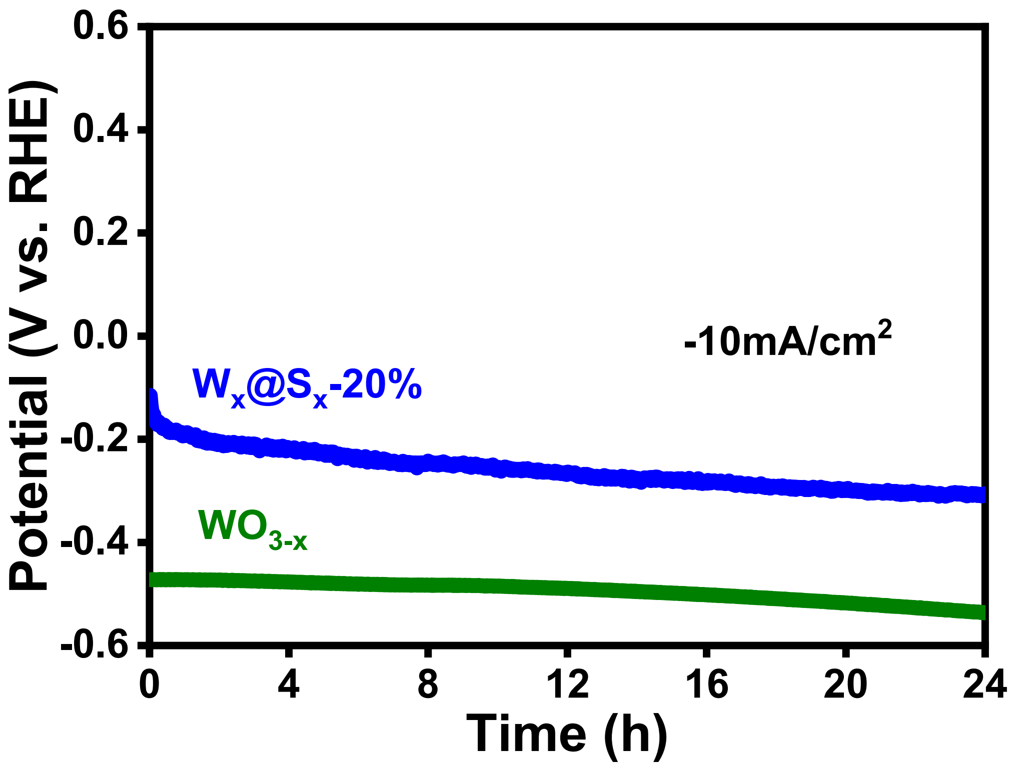


**Figure S1.** CP curves of $WO_{3-x}$ and $W_x@S_x$-20% samples.

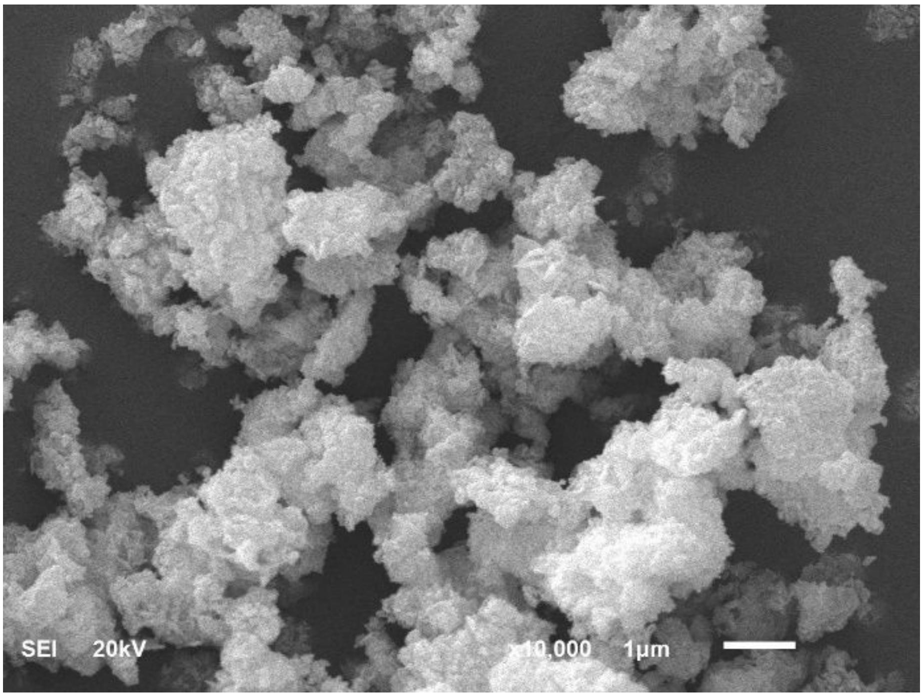


**Figure S2.** SEM micrographs of $W_x@S_x$-20% sample recorded after the stability test.

**Table S1.** A comparison of the HER activities of various electrocatalysts in the alkaline electrolyte.

| Catalysts | Overpotential (mV) (10 mA/cm$^2$) | Tafel Slope (mV/dec) | Conducting substrate / Catalyst loading (mg/cm$^2$) | Reference |
|---|---|---|---|---|
| $W_x@S_x$-20% | 191 | 61.9 | GCE / 0.198 | This work |
| $WO_3/CuO$ | 442 | 67 | CFC | [1] |
| $WO_3$ | 219 | 102 | NF | [2] |
| $WO_3/ZrO_2$ | 200 | 94 | NF | [2] |
| CoS/Ag | 284 | 70 | GCE | [3] |
| $Ru/WO_3$ | 320 | - | GCE | [4] |
| $WS_2/WO_3$ | 395 | 50 | GCE / 0.14 | [5] |
| NiWS | 201 | 93 | GCE / 0.24 | [6] |
| Pt/CdS | 103.5 | 98.5 | GCE | [7] |
| $CdS@Ni_3S_2$ | 367 | - | GCE | [8] |
| $CdS@Cu_2S$ | 447 | - | GCE | [8] |
| $NiS_2/CC$ | 243 | 104 | CC | [9] |
| $Ni_3S_2$ NWs | 200 | 141 | NF | [10] |
| $MoS_2/TiC$ | 238 | - | GCE | [11] |
| $Ni/Mo_2C$-PC | 179 | 101 | GCE / 0.25 | [12] |

NF = Nickel foam; GCE = Glassy carbon electrode; CFC = Carbon fiber cloth; CC = Carbon cloth

## References


(1) Katubi, K. M.; Warsi, A. Z.; Aziz, F.; Khattak, Z. A. K.; Warsi, M. F.; Al-Buriahi, M. S.; Alrowaili, Z. A.; Yousaf, S. Tungsten Oxide-Copper Oxide Supported on Reduced Graphene Oxide as a Proficient Electrocatalyst with Enhanced Hydrogen Evolution Efficiency in an Alkaline Media. *Current Appl Phys* **2023**, *51*, 80–90.

(2) Wei, J.; Wang, J.; Guo, W.; Tang, H.; Li, J. Tailoring the Acidity of $WO_3/ZrO_2$ to Regulate the Energy Barrier of Water Dissociation in Alkaline Hydrogen Evolution. *Chem Eng J* **2023**, *460*, 141783.

(3) Pataniya, P. M.; Patel, V.; Sahatiya, P.; Late, D. J.; Sumesh, C. K. Hydrogen Evolution Reaction in Acidic and Basic Medium on Robust Cobalt Sulphide Electrocatalyst. *Surfaces Interfaces* **2022**, *34*, 102319.

(4) Xie, X.; Fan, Y.; Tian, W.; Zhang, M.; Cai, J.; Zhang, X.; Ding, J.; Liu, Y.; Lu, S. Construction of $Ru/WO_3$ with Hetero-Interface Structure for Efficient Hydrogen Evolution Reaction. *J Energy Chem* **2023**, *83*, 150–157.

(5) Shang, X.; Rao, Y.; Lu, S.-S.; Dong, B.; Zhang, L.-M.; Liu, X.-H.; Li, X.; Liu, Y.-R.; Chai, Y.-M.; Liu, C.-G. Novel $WS_2/WO_3$ Heterostructured Nanosheets as Efficient Electrocatalyst for Hydrogen Evolution Reaction. *Mater Chem Phys* **2017**, *197*, 123–128.

(6) Premnath, K.; Madhavan, J.; Prasad, S.; Aljaafreh, M. J.; AlSalhi, M. S.; Loke, S. P. Ultra-Efficient, Low-Cost and Carbon-Supported Transition Metal Sulphide as a Platinum Free Electrocatalyst towards Hydrogen Evolution Reaction at Alkaline Medium. *Int J Hydrogen Energy* **2022**, *47* (100), 41974–41983.

(7) Fu, Y.; Li, J.; Li, J. Photo-Improved Hydrogen Evolution Reaction Activity of the Pt/CdS Electrocatalyst. *Prog Nat Sci Mater Int* **2019**, *29* (4), 379–383.

(8) Sun, M.; Guan, X.; huang, Q.; Gao, Q.; Zhang, S.; Cai, X.; Fang, Y.; Peng, F.; Yang, S. CdS@$Ni_3S_2$/$Cu_2S$ Electrode for Electrocatalysis and Boosted Photo-Assisted Electrocatalysis Hydrogen Production. *Sep Purif Technol* **2023**, *319*, 124085.

(9) Tang, C.; Pu, Z.; Liu, Q.; Asiri, A. M.; Sun, X. $NiS_2$ Nanosheets Array Grown on Carbon Cloth as an Efficient 3D Hydrogen Evolution Cathode. *Electrochim Acta* **2015**, *153*, 508–514.

(10) Ouyang, C.; Wang, X.; Wang, C.; Zhang, X.; Wu, J.; Ma, Z.; Dou, S.; Wang, S. Hierarchically Porous $Ni_3S_2$ Nanorod Array Foam as Highly Efficient Electrocatalyst for Hydrogen Evolution Reaction and Oxygen Evolution Reaction. *Electrochim Acta* **2015**, *174*, 297–301.

(11) Yu, M.; Zhao, S.; Feng, H.; Hu, L.; Zhang, X.; Zeng, Y.; Tong, Y.; Lu, X. Engineering Thin $MoS_2$ Nanosheets on TiN Nanorods: Advanced Electrochemical Capacitor Electrode and Hydrogen Evolution Electrocatalyst. *ACS Energy Lett* **2017**, *2* (8), 1862–1868.

(12) Yu, Z. Y.; Duan, Y.; Gao, M. R.; Lang, C. C.; Zheng, Y. R.; Yu, S. H. A One-Dimensional Porous Carbon-Supported Ni/$Mo_2C$ Dual Catalyst for Efficient Water Splitting. *Chem Sci* **2017**, *8* (2), 968–973.
